# Citizen scientists mapping the United Kingdom's and Republic of Ireland's flat flies (louse flies) (Diptera: Hippoboscidae) reveal a vector's range shift

**DOI:** 10.1111/mve.12795

**Published:** 2025-02-07

**Authors:** Denise C. Wawman

**Affiliations:** ^1^ Edward Grey Institute of Field Ornithology, Department of Biology University of Oxford Oxford UK

**Keywords:** birds, climate change, ectoparasites, modelling, prediction, range shift, vectors

## Abstract

Changes in climate may cause changes in the ranges, phenology and interactions of insects with other species and lead parasites to switch host species. A study of louse (flat) flies in the United Kingdom, Republic of Ireland and Isle of Man, in which licensed bird ringers acting as citizen scientists collected ectoparasites that left birds during ringing, showed recent range shifts of several species. The Common or Bird Louse Fly *Ornithomya avicularia* (Linnaeus, 1758), a vector of *Haemoproteus* sp. and trypanosomes, has undergone a major northwards range expansion of over 300 km in the United Kingdom (UK) since the 1960s. The Finch Louse Fly *Ornithomya fringillina* (Curtis, 1836) has also expanded its range over 300 km northwards and 400 km westwards into the Island of Ireland, and the Swallow Louse Fly *Ornithomya biloba* (Dufour, 1827) is now established in Wales and Southern England. The Grouse Louse Fly *Ornithomya chloropus* (Bergroth, 1901) has undergone a range contraction at lower altitudes and on the southern edge of its range. Other species of louse fly were detected: *Crataerina pallida* (Latreille, 1812), *Stenepteryx hirundinis* (Linnaeus, 1758), *Pseudolynchia garzettae* (Rondani, 1879) and *Icosta minor* (Bigot, 1858). Some generalist species have shifted their phenology, whereas the more specialist nest parasites of migrant birds have not, as the arrival and breeding dates of their hosts have not changed. The range changes of the generalist species of these ectoparasites may have implications for bird health, especially if they switch to new host species as their ranges shift.

## Graphical abstract

- The Common or Bird Louse Fly *Ornithomya avicularia,* a vector of *Haemoproteus* sp. and trypanosomes, has undergone a major northwards range expansion in the United Kingdom since the 1960s.
- The Finch Louse Fly *Ornithomya fringillina* has also expanded its range northwards and westwards, and the Swallow Louse Fly *Ornithomya biloba* is now established in Wales and Southern England.
- The range of the Grouse Louse Fly *Ornithomya chloropus* is contracting at lower altitudes at the southern edge of its range.

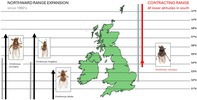

## INTRODUCTION

Changes in climate, such as those in temperature and seasonality, may affect the ranges, phenology and interactions of insects with other species (Harvey et al., 2023). Range changes are often related to physiological constraints such as thermal limits (Weaving et al., 2023), as well as changes in interactions with other species, in both food webs, with effects on their ability to find suitable food (Bartley et al., 2019), and with competitors (Gilman et al., 2010). While generalist species may be able to change their position in a food web quickly (Bartley et al., 2019), specialist species are generally slower to adapt (Menéndez et al., 2006). Nonstandardised methods of historic data collection complicate the analyses of range shifts (Guzman et al., 2021), but there are few long‐term datasets for parasitic arthropods, even for species such as ticks that are known to be important disease vectors (Nuttall, 2021).

The Hippoboscidae are a family of haematophagous ectoparasites of birds and mammals. The species found on birds are known as louse flies in most of the world but are often referred to as flat flies in the United Kingdom (UK), Republic of Ireland (ROI) and Isle of Man (Hutson, 1984), an area collectively historically referred to as the British Isles, and referred to hereafter as ‘the region’. In the UK, there are eight breeding species of flat fly (Hutson, 1984; Wawman, 2024), three of these are generalists, although with some niche separation according to host size and local environmental factors (Hill, 1962a; Lehikoinen et al., 2021). These three generalists are in the genus *Ornithomya* species group ‘a’: *O. avicularia* (Linnaeus, 1758), *O. chloropus* (Bergroth, 1901) and *O. fringillina* (Curtis, 1836) (Dick, 2018). A fourth member of the genus, the relatively recent UK colonist, *O. biloba* (Dufour, 1827), is in group ‘b’ and has a preference for Swallow *Hirundo rustica* (Linnaeus 1758) as its main host but can be found on other hirundines (Wawman, 2024). Another two fully flighted species have bred in the UK, *Pseudolynchia canariensis* (Macquart 1839), on feral pigeons *Columba livia domestica* (Gmelin, 1789), and *P. garzettae* (Rondani, 1879) on Nightjar *Caprimulgus europaeus* (Linnaeus, 1758) (Wawman, 2024). A further two species are flightless nest parasites: *Crataerina pallida* (Latreille, 1812) prefers to parasitise Swift *Apus apus* (Linnaeus 1758) but may be found on hirundines, and *Stenepteryx hirundinis* (Linnaeus, 1758) (*Crataerina hirundinis* [Linnaeus, 1758]) is found on hirundines, especially on the House Martin *Delichon urbicum* (Linnaeus, 1758) (Hutson, 1984). In Ireland, only five species of louse fly are known to breed: *O. avicularia*, *O. chloropus*, *O. fringillina*, *C. pallida* and *S. hirundinis* (O'Connor & Sleeman, 1987; Smiddy & Sleeman, 2004).

Louse flies reproduce by adenotrophic viviparity. Fertilised eggs are released one at a time into the female's uterus, where the larvae hatch and are fed from a special milk gland. When a third instar larva is mature, parturition occurs and the larva's exoskeleton hardens to form a puparium (Baker, 1967). Flightless females in the genera *Crataerina* and *Stenepteryx* leave their avian hosts to deposit their larvae in their hosts’ nests (Hutson, 1984). Females capable of flight in the genus *Ornithomya* may give birth while on their hosts or leave them when they are resting to find a suitable site to larviposit (Corbet, 1956; Hutson, 1984), and female *P. canariensis*, kept on captive domestic pigeons, were observed to deposit their larvae away from both their hosts and their nest material in darkened corners or under objects (Coatney, 1931). The well‐established species in the region undergo a winter diapause (Corbet, 1956; Hill, 1963; Walker & Rotherham, 2010b), but the recent UK colonists (Wawman, 2024) have shorter lifecycles: *P. canariensis* emerges from puparia after only 25–31 days when kept at ‘laboratory temperatures’ (Coatney, 1931); *O. biloba* has both diapausing and non‐diapausing generations (Kennedy et al., 1975).

Adults, and thus the eggs and larvae inside females, are protected against environmental extremes when on their homeothermic avian hosts but free‐flying adults and overwintering puparia may be exposed to harmful temperature, humidity and precipitation. The ability of flighted adults to find hosts maybe affected by wind speed, and in Poland, counts of the ked *Lipoptena fortisetosa* (Maa, 1965) were negatively correlated with increasing wind speed (Gałęcki et al., 2020). In common with the Hippoboscids found on birds, the puparia of the Deer Ked *Lipoptena cervi* (Linnaeus, 1758) undergo a winter diapause off their hosts and have been shown to freeze at −26°C during diapause, −20°C as developing puparia and −21°C as adults (Harkonen et al., 2012). High temperatures (20°C) during the winter diapause cause a deterioration in the immune‐based encapsulation response of diapausing *L. cervi* (Kaunisto et al., 2015). Little is known about the thermal tolerance of louse flies in the region but prolonged temperatures of 13°C or lower, or over 37°C were lethal to the puparia of *P. canariensis*, and colony survival was best between 24.6 and 30°C (Klei & Degiusti, 1975). Adult *C. pallida* successfully emerge from puparia in greater numbers at temperatures of 27–36°C—the temperature range in Swift nests during incubation—although smaller numbers emerged at lower temperatures, and none emerged when they were kept at 4–14°C to simulate winter temperatures (Walker & Rotherham, 2010a).

Thompson reported the presence of *S. hirundinis* in the British Isles from May to October, with numbers reaching a peak in June and *C. pallida* from June to August with maximum numbers in July (Thompson, 1953). Unfortunately, he did not distinguish between *O. chloropus* and *O. fringillina*, but he reported *O. avicularia* as mainly present from May to September, with a few flies in October, and November, and a peak in August (Thompson, 1954), and Hill reported this species as present from June to late November with a peak in August (Hill, 1963). *Ornithomya fringillina* was found from early July to late September at several sites, and *O. chloropus* (reported as *O. lagopodis* [Sharp, 1907]) was seen from July to September in Fair Isle (Hill, 1963). Corbet reported *O. chloropus* (as *O. fringillina* before the *Ornithomya* species were revised and the specimens reviewed [Hill, 1962b]) as present from June to mid‐August, and occasionally as late as October on Fair Isle (Corbet, 1956).

Hill reported that *O. avicularia* was, other than accidental records on migrant hosts from Scandinavia, absent from Scotland and the surrounding islands (Hill, 1962a). Whereas earlier authors (Smart, 1939) believed that *O. fringillina* was rare and restricted to south and south‐east England, Hill reported it as far north as Lancashire, with a continental distribution only as far north as Southern Sweden and Denmark (Hill, 1962a). *Ornithomya fringillina* was first reliably identified from the Island of Ireland in 1982 (Smiddy & Sleeman, 2004). *Ornithomya chloropus* was distributed in areas favoured by the Red Grouse *Lagopus lagopus scotica* (Latham, 1787), on higher ground bounded by a line that, in the early 1960s, correlated with the ‘61°F July isotherm’ (~16°C) and suggested it was a glacial phase survivor, whereas *O. fringillina*'s and *O. avicularia*'s distributions suggested that they were post‐glacial invaders (Hill, 1962a). Other earlier research on the distribution of *Ornithomya* spp. in the region (Thompson, 1954) cannot be compared with records using the current taxonomy because only two species of *Ornithomya* were recognised, instead of three. Suitable avian hosts of the generalist species are present throughout the UK and are unlikely to restrict their ranges.

Thompson suggested that *C. pallida* and *S. hirundinis* would be found throughout the breeding ranges of their hosts, although he had no records from collectors in Scotland and northern England for *C. pallida* or for the northern half of Scotland for *S. hirundinis* (Thompson, 1953). *Ornithomya biloba* was first recorded as a vagrant in the UK in 1964, with only four records up until 2007 (Lloyd‐Evans, 1967; Wawman, 2024). Its main host, the Barn Swallow, is also found throughout the UK, with the exception of Central London and the highest parts of the Scottish Highlands (Balmer et al., 2013).

Changes in the ranges of UK Hippoboscidae are expected with climate change, as many species of insect have expanded their ranges towards higher latitudes (Harvey et al., 2023) and range shifts have been seen in other Hippoboscids in Europe. For example, *Lipoptena fortisetosa* expanded its range northwards from the Czech republic, where it was first found in 1967, to reach Estonia in 2014 (Kurina et al., 2019), and *L. cervi* invaded Northern Europe to reach Finland (Kaunisto et al., 2011).

Parasite range changes may have consequences for its hosts, especially when it is a competent vector of microparasites. As a group, the Hippoboscidae are known to harbour a range of disease causing organisms although in many cases, their status as vectors is not proven (Bezerra‐Santos & Otranto, 2020). Of the long established UK species, *O. avicularia* is a proven vector of *Haemoproteus* spp. (Baker, 1963) and trypanosomes in the UK, although the latter was only transmitted if the flies were ingested by the host (Baker, 1956). Several studies have also suggested that *Leucocytozoon* sp. may be transmitted by *Ornithomya* spp. that are present in the UK, but having carefully reviewed all available evidence, Baker felt that it was not conclusive (Baker, 1967). In other countries, trypanosomes have been detected in *O. avicularia*, *O. chloropus* and *O. fringillina* but not in *C. pallida* (Santolíková et al., 2022) and several species of *Babesia* have been detected in *O. avicularia* (Čisovská Bazsalovicsová et al., 2023). Of the recent UK colonists, *P. canariensis* is a vector of *Haemoproteus columbae* (Baker, 1967; Cepeda et al., 2019) and trypanosomes were detected in 18.7% of *O. biloba* in a study in Czechia (Santolíková et al., 2022). *Babesia canis* has also been detected in *O. biloba* (Čisovská Bazsalovicsová et al., 2023). *Rickettsia belii* and *Rickettsia monacensis* have been detected in *C. pallida* (Cerutti et al., 2018), but a study failed to detect *Haemoproteus* spp. in either this fly or its host the Swift in Italy (Ilahiane et al., 2023).

The Mapping the UK's Flat Flies Project is an ongoing citizen science project in which licensed British Trust for Ornithology (BTO) bird ringers are asked to collect flat flies that leave birds during their normal ringing sessions, with the aim of determining which species are present in the region, their geographical and host ranges, and the phenology of the species. It has expanded to include Hippoboscidae of all species, across the region made up of the UK, ROI and Isle to Man. This paper considers the geographical ranges and phenology of the louse fly species as revealed by data collected from June 2020 to May 2024. The presence of new species breeding in the UK has been previously discussed (Wawman, 2024), and the host–parasite associations revealed will be the topic of a further study.

## METHODS

The Mapping the UK's Flat Flies Project began as a small pilot study in 2020 and is an ongoing study run as part of the UK Hippoboscidae and Nycteribiidae Recording Scheme. BTO volunteer bird ringers were recruited via social media groups, articles in the BTO's ‘Lifecycle’ magazine and personal communications and were asked to collect louse flies that were seen leaving birds during their normal ringing activities, as current UK animal welfare regulations prevent the use of methods such as ‘dust‐ruffling’ using insecticidal powders (Walther & Clayton, 1997) or a Fair Isle Apparatus (Williamson, 1954). Participants were sent recording forms, tubes and 70% ethanol and asked to return any flies with the metadata at the end of the season.

Volunteers were asked to collect the following metadata: site name, Ordnance Survey Grid Reference, altitude and, if known, the host species, the number of flies both seen on and collected from each host and its age, sex and moult status, using BTO ringing codes (Redfern & Clark, 2001).

Keds were collected by bird ringers, entomologists and interested members of the public, via both the main mapping project and the national recording scheme.

The flies were identified to species, under a binocular microscope, following a published key and descriptions of flat flies in the British Isles (Hutson, 1984). Additional information for rarer species was sourced from a range of publications (Hutson, 1981a; Maa, 1964, 1966, 1969; Maa & Petersen, 1987).

The grid references and site altitudes supplied in the metadata, that were used for the initial analyses, were checked on the website Curaera (www.cucaera.co.uk/grp last accessed 27 May 2024).

The data were analysed in R version 4.2.1 (R Development Core Team, 2022). The packages *dplyr* (Wickham et al., 2023) and *lubridate* (Grolemund & Wickham, 2011) were used to process the data prior to analysis. Graphs were made in base R, and basic maps were plotted using packages *maps* (Becker et al., 2022) and *mapdata* (Becker et al., 2018), with the package *scales* (Wickham & Seidel, 2022). Kernel densities were calculated using the packages *adehabitatHR* (Calenge, 2006) and *rgdal* (Bivand et al., 2023). Bioclimatic data and species data were formatted into rasters and manipulated in R using the package *raster* (Hijmans, 2023). The maps of previously published ranges were replotted using the functions on the website MapChart (https://www.mapchart.net/uk.html, last accessed 10th June 2024). Binomial glms, comparing pairs of the *Ornithomya* sp., and *O. chloropus* with all the other *Ornithomya* in the study were run in R (family = binomial, link = cloglog) with the package *arm* used to validate the models (Gelman & Su, 2022).

Bioclimatic data, for 2022, the middle year of the main study, and most recent year available, were sourced from the HadUK‐Grid data (Met Office et al., 2023). The UK altitude, as the mean altitude for each 1 km^2^ square, was taken from Intermap (Intermap. NERC Earth Observation Data Centre, 2009), land cover from LCM 2007 (Morton et al., 2011), protected area status from the UNEP‐WCMC and IUCN Protected Planet Report 2020 (UNEP‐WCMC and IUCN, 2020) and calcareous bedrock from the British Geological Survey's parent material model (British Geological Survey, 2024; Lawley & Rawlins, 2014). The bioclimatic and land cover variables were used in the species distribution modelling, and the rationale for their inclusion are shown in Table 1. It was not possible to obtain data on the same grid scale for some of these variables for the ROI and those which were available were on a different spatial grid and geographical projection, so, as the area was only sparsely sampled, these data were not included in the most complex level of modelling.

**TABLE 1:** Variables used in the Maxent SDMs and the rationale for choosing them and hypotheses to be tested.

Description	Year	Source	Rationale for inclusion in initial models/hypotheses to be tested	Reason for exclusion from final models
Latitude			Initial data exploration suggested this was important, frequently found to be important in similar studies	
Longitude			Initial data exploration suggested this was important, frequently found to be important in similar studies	
Altitude (m above sea level)	2009	Intermap (2009)	Initial data exploration suggested this was important, frequently found to be important in similar studies	
Calcareous bedrock (% cover per square km)	2014	British Geological Survey	Soil pH may affect survival of invertebrates, relevant to species where puparia may be on the ground	Not found to be relevant
Ground frost annual (number of days)	2022	Met Office HadUK‐Grid	Prolonged low temperatures may affect survival more than shorter cold spells, potentially represented by minimum temperatures	
Minimum air temperature seasonal (°C)	2022	Met Office HadUK‐Grid	May be lower than lower critical thermal range	Highly correlated with other temperature measures
Maximum air temperature seasonal (°C)	2022	Met Office HadUK‐Grid	May exceed higher critical thermal range	
Mean air temperature annual (°C)	2022	Met Office HadUK‐Grid	May be a suitable proxy for both maximum and minimum temperature which are likely to be highly correlated and allow a reduction in the number of variables	Highly correlated with other temperature measures
Relative humidity annual (hPa)	2022	Met Office HadUK‐Grid	Desiccation may decrease survival of puparia	
Wind speed at 10 m—annual—(m s^−1^)	2022	Met Office HadUK‐Grid	May affect flighted flies’ ability to find a host	
Precipitation seasonal (mm)	2022	Met Office HadUK‐Grid	Possibility of puparia drowning, rain may affect flighted species ability to find a host	
Urban land cover (% cover per square km)	2007	2007 UKCEH land cover map	Some species (*Stenepteryx hirundinis*, *Crataerina pallida* and *Ornithomya biloba*) are associated with hosts that nest in buildings. To check for spatial bias in data. Possible urban heat island effects	
Protected area status (proportion per square km)	2020	UNEP‐WCMC and IUCN (2020)	To check for spatial bias in data	Not found to be relevant

Abbreviation: SDM, species distribution modelling.

As bioclimatic data were not available for some smaller islands and coastal areas with less than 50% land within a 1‐km grid square, these were dealt with in one of two ways. Where there was land immediately adjacent to the site (for example, Portland Bird Observatory and Calf of Man Bird Observatory), the bioclimatic data for the most similar adjacent square (north and west respectively) were used. Where there was no adjacent Ordnance Survey Grid Square with available data (for example, Skokholm Bird Observatory), that of the nearest similar land was used (Skomer Island).

Maximum entropy species distribution modelling (Maxent SDM) was performed using the program Maxent version 3.4.1 (Phillips et al., 2024) using the default settings. After removal of highly correlated variables, the best models were chosen on the basis of the area under the receiver operator curve (AUC) and by visual inspection of the maps produced for anomalies. Variables that did not contribute to the model of each species distribution were excluded based on a combination of permutation importance, jackknife plots and response curves. The data were checked for the presence of spatial bias in favour of urban areas, which is known to occur in many citizen science datasets (Bowler et al., 2022; Sumner et al., 2019), and protected areas (Bowler et al., 2022) by modelling the locations of all flies collected against both urban land cover and protected land cover. The maps published here were produced from the mean of five replicates using the final set of variables.

Flies raised from puparia were excluded from the analysis of phenology as these may have emerged earlier than they would have normally due to being kept indoors.

## RESULTS

Total of 3506 Hippoboscids, of 11 species, were received from over 170 individual bird ringers and entomologists, bird observatories and ringing groups up until the date of the analysis in May 2024. These came from 350 10‐km grid squares across the UK, Isle of Man and ROI. The flies were collected over a 22‐year period from 2002 to 2024, with most collected during the main study period from June 2020 to May 2024. The totals included eight species of louse fly and all three UK species of ked. The only UK breeding species that was missing was the Pigeon Louse Fly, *P. canariensis*, as the BTO bird ringing scheme does not include feral pigeons. The total numbers of each species received are provided in Table 2, with a breakdown of the number of 1‐km and 10‐km grid squares from which they were collected. A map of all the sites at which specimens were collected can be found in the Figure S1.

**TABLE 2:** Total number Hippoboscids collected (louse/flat flies and keds), number of 1 km Ordnance Survey gird squares square (UK and Isle of Man) that were included in the Maxent species distribution models and total number of 10 km grid squares (UK, Isle of Man and Republic of Ireland) where each species was reported. Some specimens were only supplied with a 10‐km grid reference. *Icosta minor is a vagrant, while the other species are known to breed in the region. It was not possible to identify one damaged *Ornithomya* sp. specimen. The keds are included for completeness although they do not form part of the main study of bird parasites and *Pseudolynchia canariensis* is included to highlight the absence of data from this study.

Species	Number of flies	Number of 1 km squares (United Kingdom only)	Total number of 10 km squares
Louse/flat flies			
Swift Louse Fly *Crataerina pallida* (Latreille, 1812)	60	22	21
*Icosta minor* (Bigot, 1858)*	1	1	1
Common or Bird Louse Fly *Ornithomya avicularia* (Linnaeus, 1758)	1438	249	217
Swallow Louse Fly *Ornithomya biloba* (Dufour, 1827)	30	13	13
Grouse Louse Fly *Ornithomya chloropus* (Bergroth, 1901)	864	136	103
Finch Louse Fly *Ornithomya fringillina* (Curtis, 1836)	848	139	123
*Ornithomya* sp.	1	1	1
Pigeon Louse Fly *P. canariensis* (Macquart, 1839)	0	0	0
Nightjar Louse Fly *Pseudolynchia garzettae* (Rondani, 1879)	3	2	2
Martin Louse Fly *Stenepteryx hirundinis* (Linnaeus, 1758)	155	30	29
Keds			
New Forest Fly *Hippobosca equina* (Linnaeus, 1758)	3	1	2
Deer ked *Lipoptena cervi* (Linnaeus, 1758)	99	21	18
Sheep Ked *Melophagus ovinus* (Linnaeus, 1758)	4	2	2
All species	3506	463	350

The Maxent SDM showed that there was no evidence of bias favouring records from urban or protected areas (Figure S2) with an AUC of 0.541 (0.5 is random, range 0 to 1.0) for urban landcover, 0.563 for protected area status and 0.604 for both urban and protected areas combined, suggesting that the overall sampling distribution of the study was close to random in relation to both human population density and protected area status. As might be expected, a kernel density plot made from the locations of all individual flies collected in the study (Figure S3) showed that collection was not even across the region, reflecting both differences in collecting effort, including poor coverage over most of the island of Ireland, and the populations of Hippoboscids.

**FIGURE 1:**
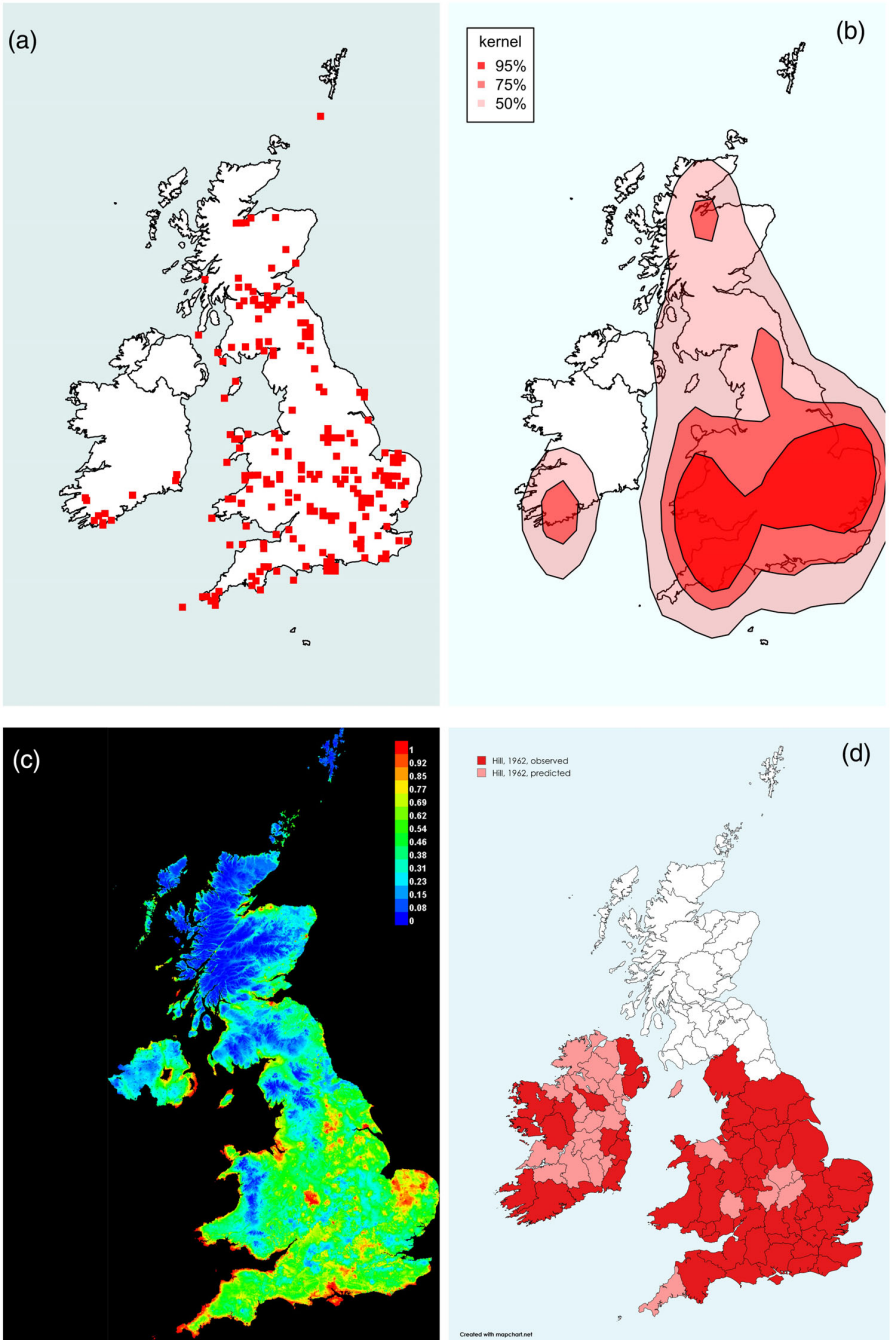
Range of the Common or Bird Louse Fly *Ornithomya avicularia* (Linnaeus, 1758). (a) Locations of each 10 km square in which *O. avicularia* was caught during the study. (b) Kernel density plot at 95% predicted probability (dark red), 75% probability and 50% probability of finding *O. avicularia* at a given location, based entirely on latitude, longitude and presence data. (c) Maxent SDM, red 100% probability of the environmental niche being suitable for *O. avicularia*, with orange, yellow and green being increasingly lower probabilities of finding it (green 50% and blue 0%). This plot was produced from five iterations of the Maxent SDM using the final list of bioclimatic variables in Table 1 combined with the presence data from the study (AUC = 0.737). (d) Hill's 1962 ranges for the *O. avicularia*, produced at county level, replotted. The darker red areas are where the species was found: paler areas where he expected it to be present. Note that Hill removed flies found on passage migrant birds from his analysis, whereas they have not been removed from the analyses in this study. AUC, area under the receiver operator curve; SDM, species distribution modelling.

**FIGURE 2:**
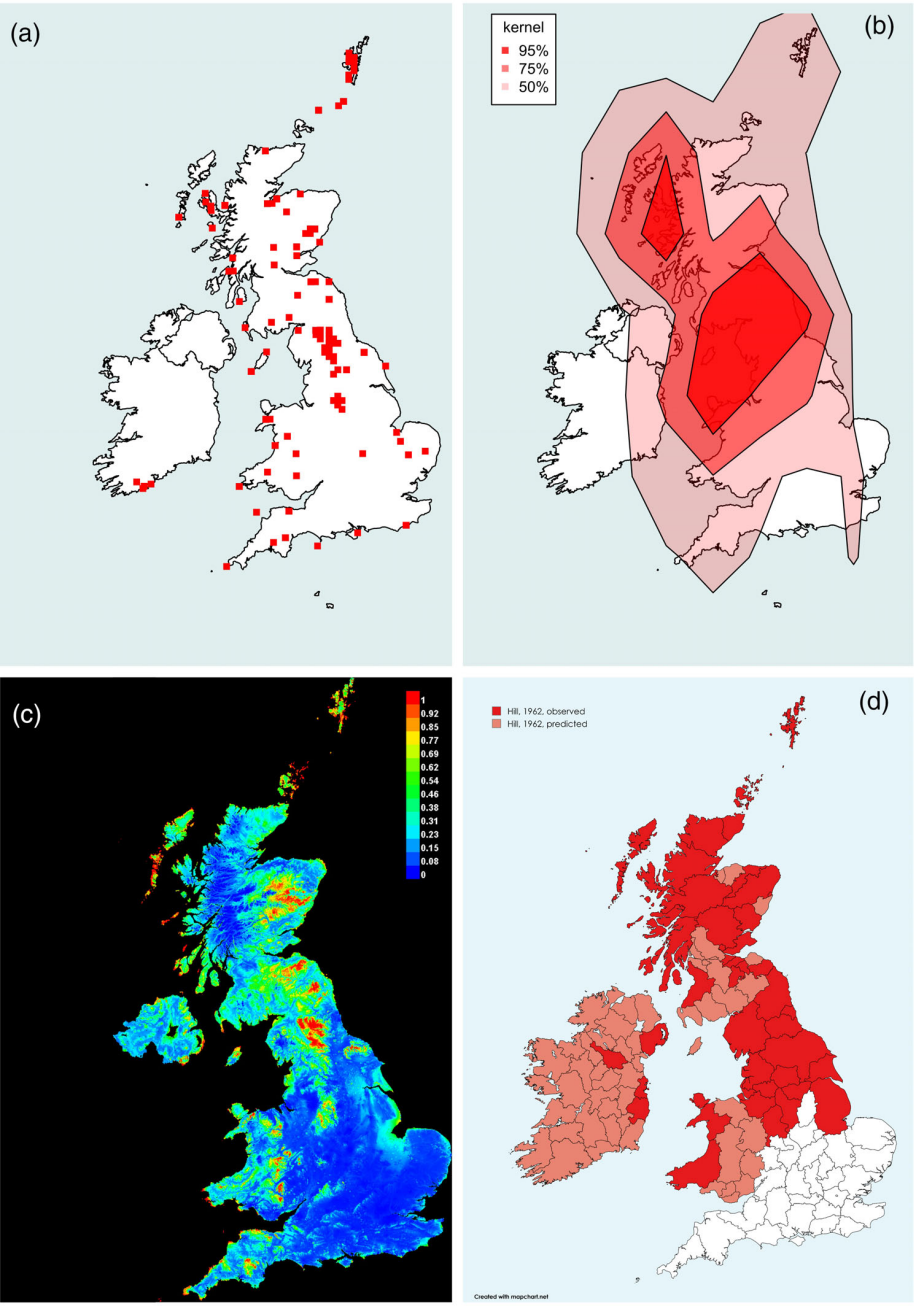
Range of the Grouse Louse Fly *Ornithomya chloropus* (Bergroth, 1901). (a) Locations of each 10 km square in which *O. chloropus* was caught during the study. (b) Kernel density plot at 95% predicted probability (dark red), 75% probability and 50% probability of finding *O. chloropus* at a given location, based entirely on latitude, longitude and presence data. (c) Maxent SDM, red 100% probability of the environmental niche being suitable for *O. chloropus*, with orange, yellow and green being increasingly lower probabilities of finding it (green 50% and blue 0%). This plot was produced from five iterations of the Maxent SDM using the final list of bioclimatic variables in Table 1 combined with the presence data from the study (AUC = 0.810). (d) Hill's 1962 ranges for the *O. chloropus*, produced at county level, replotted. The darker red areas are where the species was found: paler areas where he expected it to be present. Note that Hill removed flies found on passage migrant birds from his analysis, whereas they have not been removed from the analyses in this study. AUC, area under the receiver operator curve; SDM, species distribution modelling.

**FIGURE 3:**
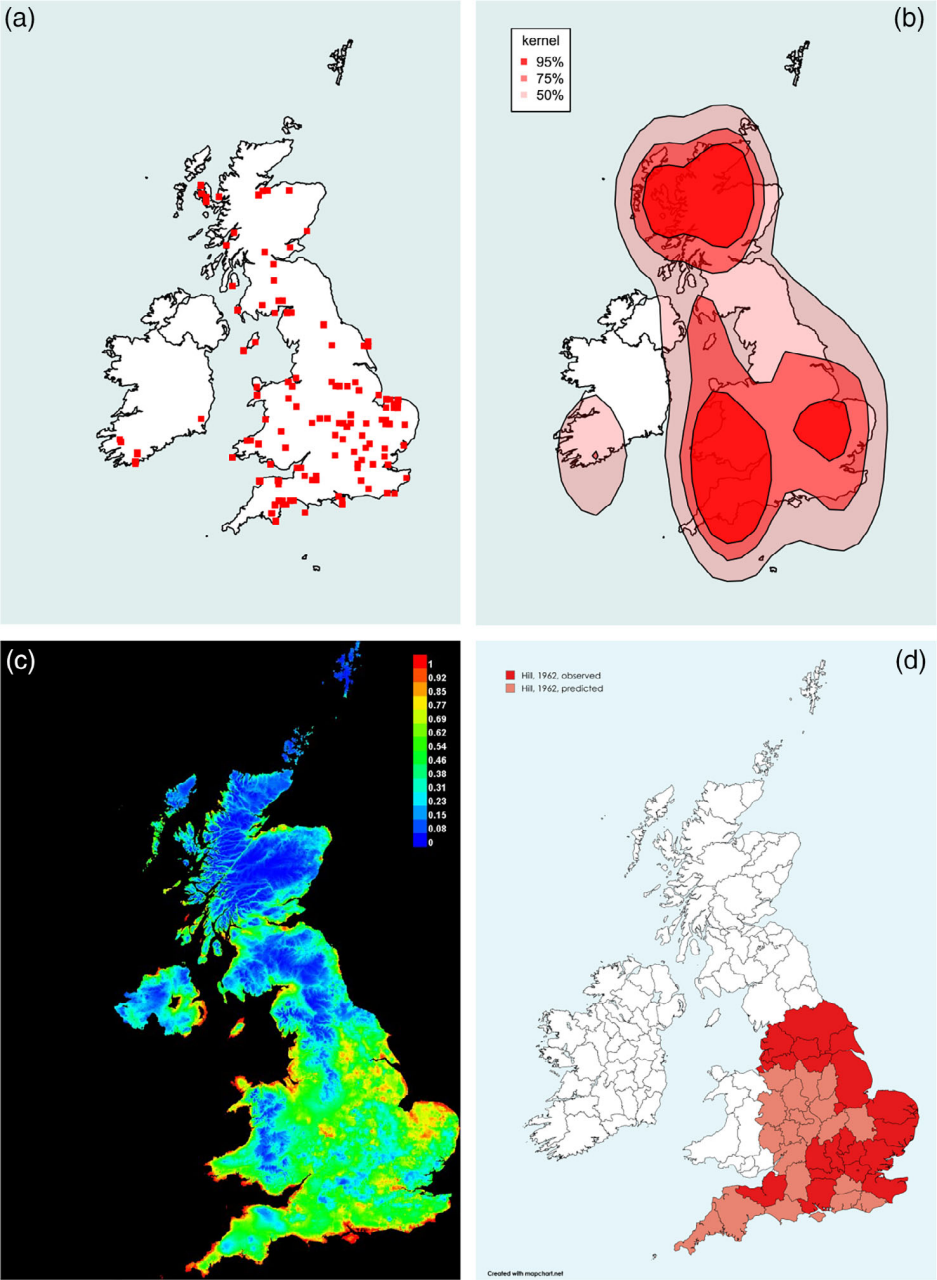
Range of the Finch Louse Fly *Ornithomya fringillina* (Curtis, 1936). (a) Locations of each 10 km square in which *O. fringillina* was caught during the study. (b) Kernel density plot at 95% predicted probability (dark red), 75% probability and 50% probability of finding *O. fringillina* at a given location, based entirely on latitude, longitude and presence data. (c) Maxent SDM, red 100% probability of the environmental niche being suitable for *O. fringillina*, with orange, yellow and green being increasingly lower probabilities of finding it (green 50% and blue 0%). This plot was produced from five iterations of the Maxent SDM using the final list of bioclimatic variables in Table 1 combined with the presence data from the study (AUC = 0.738). (d) Hill's 1962 ranges for the *O. fringillina*, produced at county level, replotted. The darker red areas are where the species was found: paler areas where he expected it to be present. Note that Hill removed flies found on passage migrant birds from his analysis, whereas they have not been removed from the analyses in this study. AUC, area under the receiver operator curve; SDM, species distribution modelling.

**FIGURE 4:**
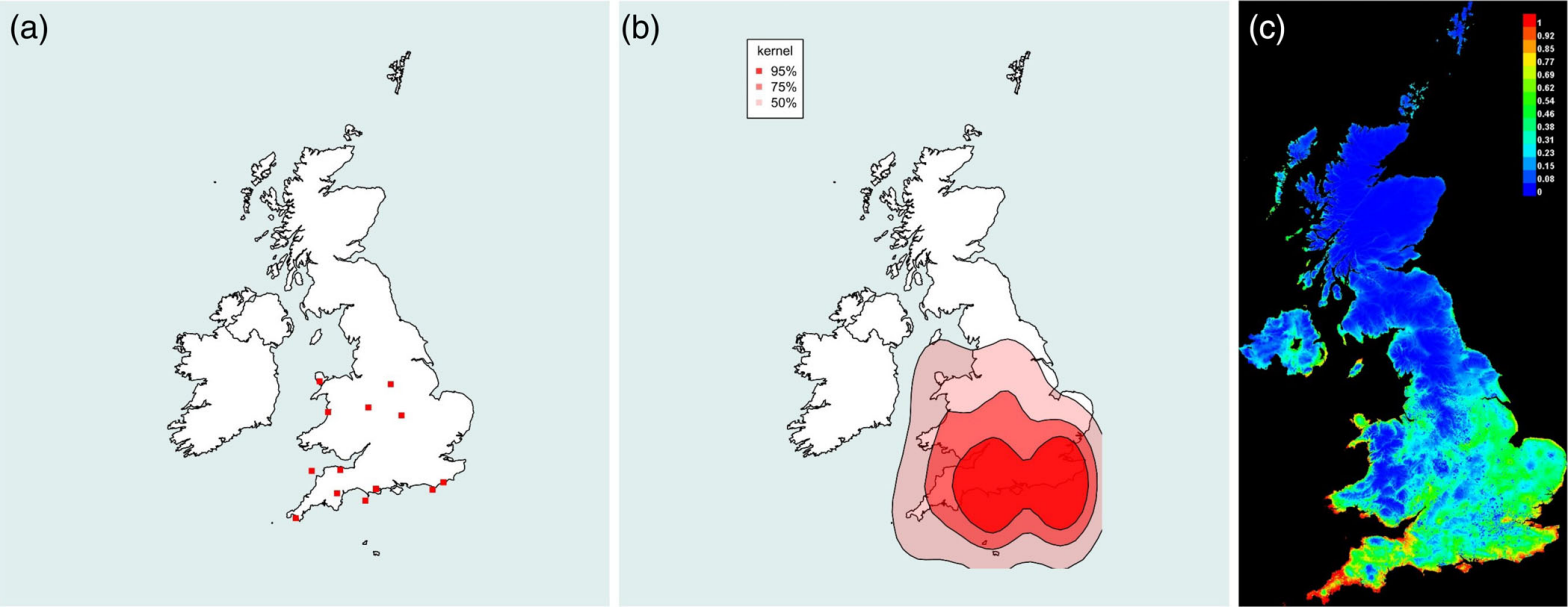
Range of the Swallow Louse Fly *Ornithomya biloba* (Dufour, 1827). (a) Locations of each 10 km square in which *O. biloba* was caught during the study. (b) Kernel density plot at 95% predicted probability (dark red), 75% probability and 50% probability of finding *O. biloba* at a given location, based entirely on latitude, longitude and presence data. (c) Maxent SDM, red 100% probability of the environmental niche being suitable for *O. biloba* , with orange, yellow and green being increasingly lower probabilities of finding it (green 50% and blue 0%). This plot was produced from five iterations of the Maxent SDM using the final list of bioclimatic variables in Table 1 combined with the presence data from the study (AUC = 0.805). AUC, area under the receiver operator curve; SDM, species distribution modelling.

**FIGURE 5:**
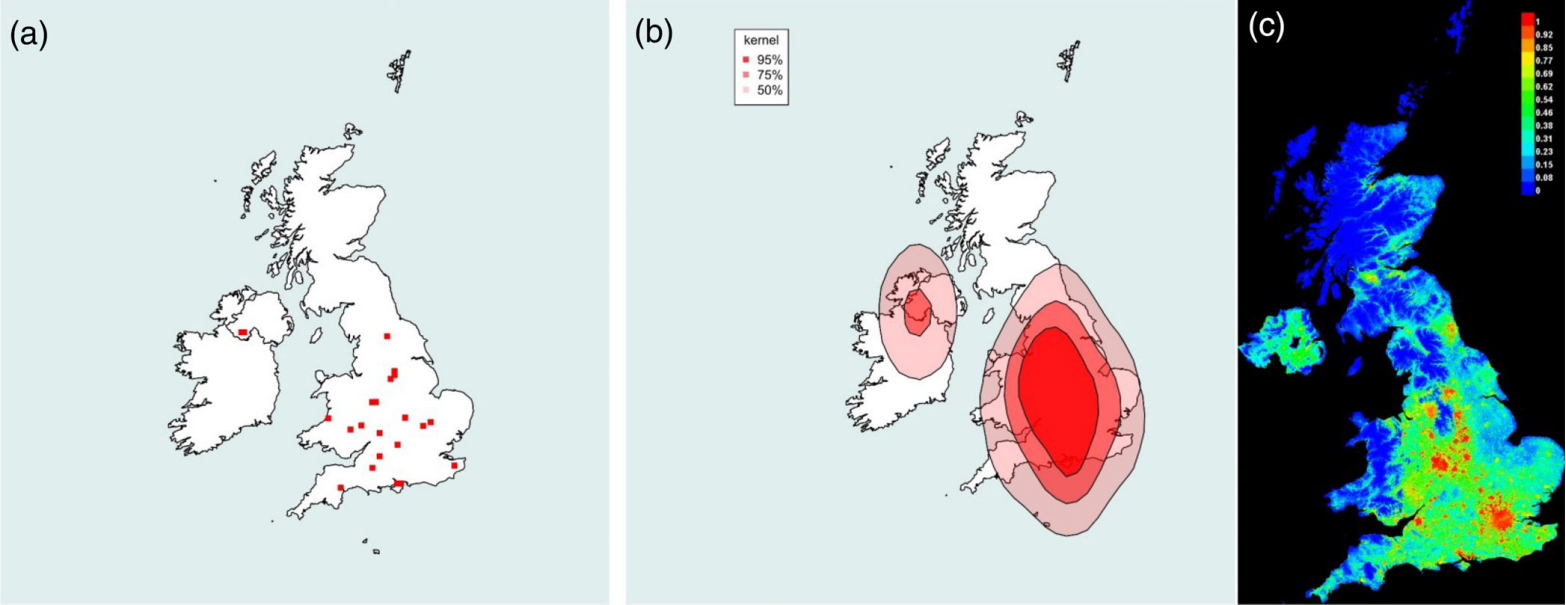
Range of the Swift Louse Fly *Crataerina pallida* (Latreille, 1812). (a) Locations of each 10 km square in which *C. pallida* was caught during the study. (b) Kernel density plot at 95% predicted probability (dark red), 75% probability and 50% probability of finding *C. pallida* at a given location, based entirely on latitude, longitude and presence data. (c) Maxent SDM, red 100% probability of the environmental niche being suitable for *C. pallida* , with orange, yellow and green being increasingly lower probabilities of finding it (green 50% and blue 0%). This plot was produced from five iterations of the Maxent SDM using the final list of bioclimatic variables in Table 1 combined with the presence data from the study (AUC = 0.807). AUC, area under the receiver operator curve; SDM, species distribution modelling.

**FIGURE 6:**
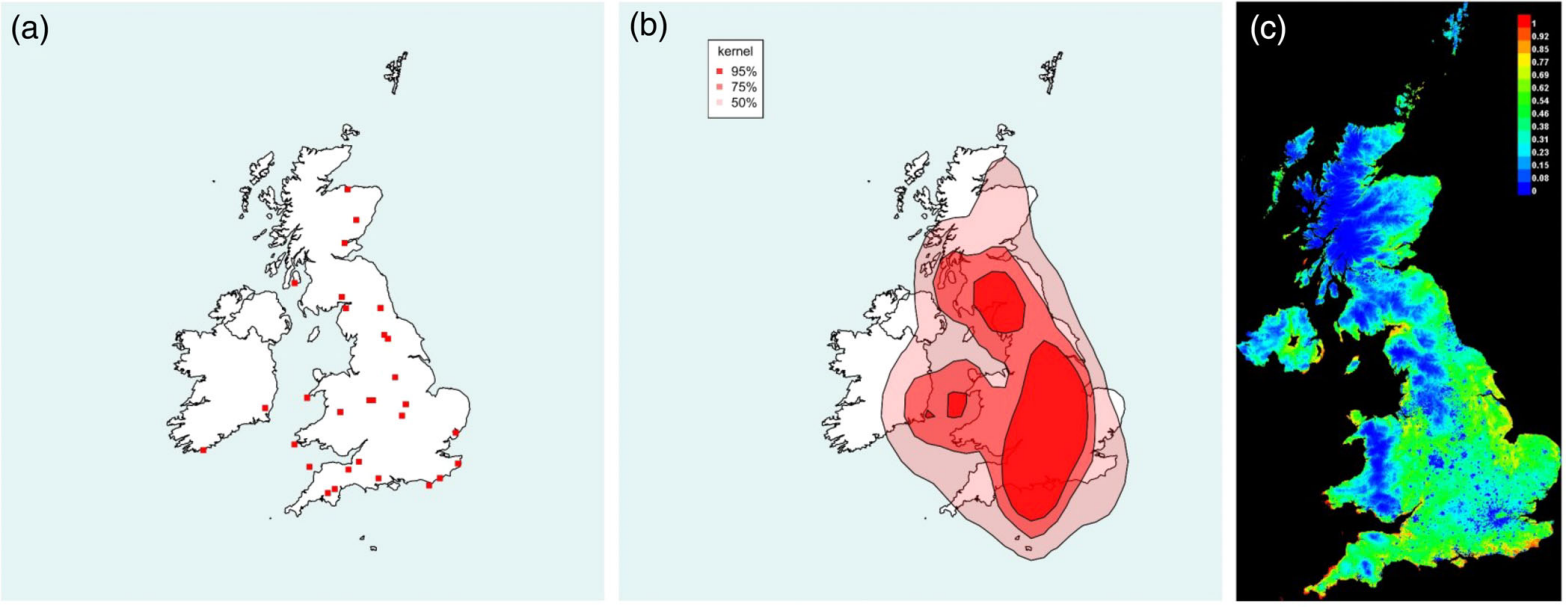
Range of the Martin Louse Fly *Stenepteryx hirundinis*. (a) Locations of each 10 km square in which *S. hirundinis* was caught during the study. (b) Kernel density plot at 95% predicted probability (dark red), 75% probability and 50% probability of finding *S. hirundinis* at a given location, based entirely on latitude, longitude and presence data. (c) Maxent SDM, red 100% probability of the environmental niche being suitable for *S. hirundinis*, with orange, yellow and green being increasingly lower probabilities of finding it (green 50% and blue 0%). This plot was produced from five iterations of the Maxent SDM using the final list of bioclimatic variables in Table 1 combined with the presence data from the study (AUC = 0.670). AUC, area under the receiver operator curve; SDM, species distribution modelling.

The distributions of each of the six most common species (*O. avicularia*, *O. chloropus*, *O. fringillina*, *O. biloba*, *C. pallida* and *S. hirundinis*) can be seen as a series of plots in Figures 1, 2, 3, 4, 5, 6, which show the location at which specimens were collected, estimates of distribution using kernel densities and Maxent SDMs, together with the previously published distributions (Hill, 1962a) for the generalist *Ornithomya* spp. Only three specimens of *Pseudolynchia garzettae* (the UK's and the region's second, third and fourth) and one *Icosta minor* (the region's and UK's fourth) were received, and this was insufficient to run a Maxent SDM with the number of variables used in the analyses.

*Ornithomya avicularia* (Figure 1) has increased its UK range approximately 300 km northwards, from the 1960s, when it was rarely found north of latitude 55°N, roughly the latitude of the Isle of Man, to reach Inverness, latitude 57.5°N. It was caught in numbers at multiple locations (Figure 1a) in the north of England and Scotland where it was not present in the 1960s (Figure 1d). The kernel density plot (Figure 1b) indicates a range covering most of Great Britain, with a concentration of records from England and Lowland Scotland, but does not take altitude and other bioclimatic variables into account. The Maxent SDM (Figure 1c) predicts suitable bioclimatic niches (shown on the map as green, through yellow, orange and red with increasing probability), corresponding to the sites at which flies were observed. It shows a likely current range throughout the UK, with the exception of the highest ground in England and Wales, and all but the lowest ground in Scotland.

*Ornithomya chloropus* (Figure 2) may have undergone a slight range contraction at lower altitudes at the southern edge of its range. The few records (Figure 2b) from southern England were mainly on suspected migrant birds near the coast—a set of records that was excluded by Hill (Hill, 1962a) (Figure 2d)—or restricted to higher ground on Dartmoor and Exmoor. *Ornithomya chloropus* was also detected on breeding birds on Skokholm Island and Lundy Island off the southern coasts of the UK. *Ornithomya chloropus* has a marked presence along The Pennines—the ridge of hills running north–south in northern England—and is present throughout Scotland. It appears from the map of sites (Figure 2a) and the kernel density plot (Figure 2c) to have been lost from the most southerly part of its former distribution (Figure 2d). The Maxent SDM (Figure 2c) predicts the highest probability of finding areas suitable for *O. chloropus* (red) is at higher altitudes in Scotland, Wales, northern and southwest England, as well as on islands around the coast.

*Ornithomya fringillina*, in contrast to *O. chloropus* (Figure 3), has, like *O. avicularia*, greatly expanded its UK range. It was mainly restricted to southern England below 54°N, an area south of a line from Blackpool to Leeds, in the 1960s (Figure 3d), but is now seen throughout lowland Scotland to latitude 57.5°N, a distance of over 350 km further north. It has also expanded its range over 400 km westwards into Wales and the ROI. The kernel density plot (Figure 3c) fails to take altitude into account, showing an unlikely distribution over higher ground in Scotland and Wales. The Maxent SDM (Figure 3c) predicts suitable environmental niches present throughout the UK, except at higher altitudes.

*Ornithomya biloba* (Figure 4) was first recorded as a vagrant in the UK in 1964. Figure 4a shows the location of sites at which it was found; Figure 4b shows the predicted range throughout southern England and all of Wales, south of latitude 53.5°N, using kernel densities; and Figure 4c shows a similar predicted range from a Maxent SDM.

*Crataerina pallida* (Figure 5) and *S. hirundinis* (Figure 6) were described in the 1950s as being present throughout the region wherever their hosts were found (Thompson, 1953). The sites at which they were recorded (Figures 5a and 6a) were widely dispersed, with the Maxent SDMs (Figures 5c and 6c) suggesting suitable habitat in all lowland areas of the UK, but showing *C. pallida* favouring urban areas and *S. hirundinis* rural areas, in common with their respective preferred avian hosts.

**FIGURE 7:**
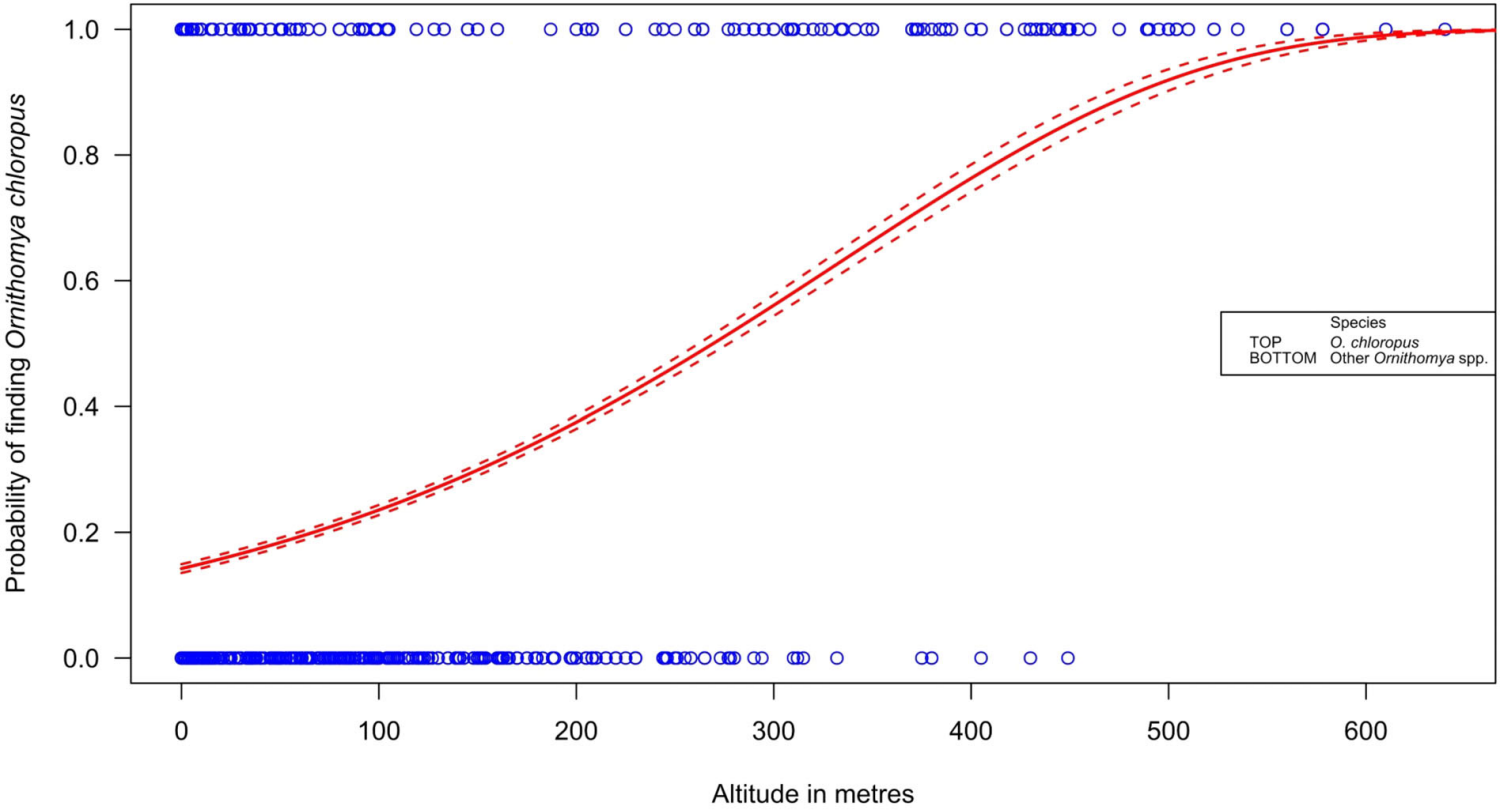
The probability of finding a species at a given altitude with standard error lines (dotted) from the output of a binomial glm comparing the altitude at which *Ornithomya chloropus* (top) is found to the that other species in the genus *Ornithomya* (*O. avicularia*, *O. fringillina* and *O. biloba* ); probability, *p* < 0.001.

Analyses of the altitude ranges of each species showed that *O. chloropus* is present at higher altitudes than the other species in the genus *Ornithomya* (Table 3; Figure 7). None of the other species were seen above 449 m, whereas *O. chloropus* was seen up to 640 m, and a binomial glm (Figure 7) predicted an increasing probability of finding *O. chloropus*, rather than other species, as altitude increases with a 100% expectation that a fly would be *O. chloropus* at 600 m and above. A boxplot of the altitude ranges of all the species can be found in Figure S4.

**TABLE 3:** The altitude ranges of the *Ornithomya* species in metres.

Species	Altitude range (m)	Mean altitude (m)	Median altitude (m)
*Ornithomya avicularia*	0–449	84.9	64.0
*Ornithomya chloropus*	0–640	185.1	80.0
*Ornithomya fringillina*	0–290	74.7	60.0
*Ornithomya biloba*	2–278	44.6	20.0
*O. avicularia* + *O. fringillina* + *O. biloba*	0–449	80.6	64.0

**FIGURE 8:**
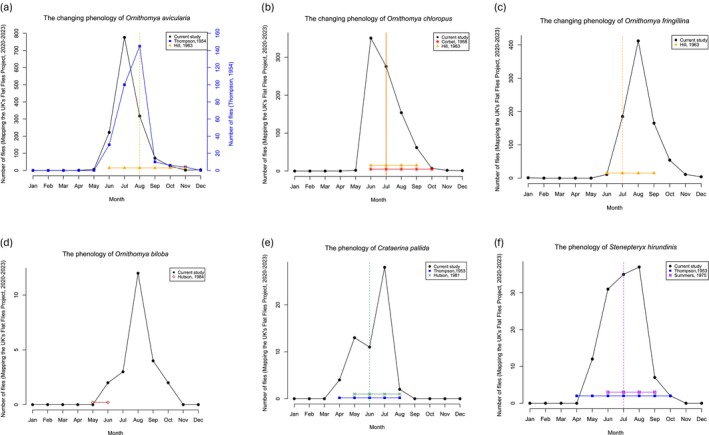
The phenology of the UK louse flies. The current study shown in black. With results of previous studies: Thompson (1953, 1954)) in blue, Hill (1963)) in orange, Corbet (1956)) in red, Summers (1975)) in purple, Hutson (1981b)) in green, and Hutson, 1981b) in brown. For most of these studies, only a range is dates and a peak, if one is available, is given, and where this is the case, the months in which the species were reported are plotted as a horizontal line close to zero, with a vertical line to show the reported peak. (a) *Ornithomya avicularia*, (b) *Ornithomya chloropus*, (c) *Ornithomya fringillina*, (d) *Ornithomya biloba*, (e) *Crataerina pallida* and (f) *Stenepteryx hirundinis*.

The numbers of flies caught per month, of the six most common species are shown in Figure 8 plotted with the results of previous studies. These show that the peaks in the numbers of both *O. avicularia* (Figure 8a) and *O. chloropus* (Figure 8b) now occur a month earlier than previously. *Ornithomya fringillina* peaks a month later (Figure 8c). *Ornithomya biloba* is now found throughout the breeding season of its host, rather than as a vagrant on a few early Swallows. The phenology of *C. pallida* and *S. hirundinis* appears unchanged from the mid‐20th century.

A comparison of the number of flies of each species in genus *Ornithomya* seen in this study with that in the 1960s (Hill, 1962a) suggests that *O. avicularia* may have replaced *O. chloropus* as the most common species across the UK (Table 4).

**TABLE 4:** Comparison of sample sizes of the *Ornithomya* species between this study and that by Hill (1962a)).

Species	Hill (*n*)	Hill (% of total)	Current study (*n*)	Current study (% of total)
*Ornithomya avicularia*	1030	29.2	1438	45.2
*Ornithomya chloropus*	2082	58.9	864	27.2
*Ornithomya fringillina*	420	11.9	848	26.7
*Ornithomya biloba*	0	0	30	0.9
Total	3532	100	3180	100

## DISCUSSION

The results of this study suggest that the ranges of *O. avicularia* and *O. fringillina* have undergone significant northward shift in the 60 years since their ranges were last mapped in the 1960s (Hill, 1962a). *Ornithomya avicularia* and *O. fringillina* were both found in significant numbers at sites over 300 km north of their previously published distributions, and Maxent SDMs predicted that there are suitable climatic niches up to the northern tip of Scotland over 100 km beyond their recorded distribution. *Ornithomya fringillina* has also colonised areas over 400 km west of its previously recorded range, in the island of Ireland, where it was first recorded in 1982 (Smiddy & Sleeman, 2004). There are suitable climatic niches for the recent colonist *O. biloba* that could allow it to become widely distributed throughout Wales and Southern England. *Ornithomya chloropus* may have undergone a slight contraction of its range at lower altitudes especially in Yorkshire and around Liverpool and Manchester, and it appears to be less common, but comparisons to this level of accuracy are difficult because Hill's data were only plotted to county level. There was insufficient data, both in terms of published maps and within the current dataset, to thoroughly explore the current ranges of *C. pallida* and *S. hirundinis*. The analyses show shifts in the phenology of the three most common species, with *O. avicularia* and *O. chloropus* reaching peaks in their populations a month earlier than previously and numbers of *O. fringillina* peaking a month later. Despite few specimens of *C. pallida* and *S. hirundinis* being received their phenology has remained fairly constant. The current study also detected differences in the proportions of the *Ornithomya* species compared with Hill (1962a), with *O. avicularia* now the most numerous species, compared with *O. chloropus* in the past.

Many species have shifted their ranges northwards since the 1960s. For example, over a 25‐year period, there was a mean northwards range shift of 31–61 km in 275 out of the 329 species studied across 16 vertebrate and invertebrate taxa in Great Britain (Hickling et al., 2006). Many of Britain's larger moths (Lepidoptera) have shifted their ranges northwards by over 200 km between the two recording periods 1968–1990 and 1992–2011 (Fox et al., 2013). British dragonflies (Odonata) have shifted their ranges northwards by a mean of 74 km, between recording periods 25 years apart (1960–1970 and 1985–1995) with the northern most edge of the range of the Common Darter *Sympetrum striolatum* (Charpentier, 1840) moving a further 346 km north (Hickling et al., 2005). Even when the longer duration between the two studies compared in this paper is taken into account, the range shifts suggested by this study would appear to be over a greater distance compared with those of most UK insects. However, other Hippoboscids, have also advanced their ranges northwards at a higher than average rate: *Lipoptena* f*ortisetosa* advanced its European range from Czechia to Estonia, a distance of over 1000 km between 1967 and 2014 (Kurina et al., 2019), and *L. cervi* expanded its range northwards by 1000 km in 50 years. While most species of bird in the UK have only shifted their ranges northwards by an estimated 0.76 km per year (Gillings et al., 2015), generalist parasites such as *O. avicularia* and *O. fringillina* are not tied to a single host species and can switch hosts to rapidly expand their ranges as conditions permit.

Adult Hippoboscids and their larvae on endothermic hosts, such as Cervids or birds, will be protected from extremes of climate during those phases of their lifecycle, but will be more vulnerable as puparia. The puparia of the nest parasites *C. pallida* and *S. hirundinis* are protected from the worst winter weather as they are in the nests of birds, usually within or attached to buildings, but unlike the flighted species, are likely to be slower to colonise a new area as they have to rely entirely on birds or crawling to get to new sites. Flighted species such as the *Ornithomya* spp. are able to colonise new areas by flying to find a host, but with the exception of *O. biloba*, which is a nest parasite, are vulnerable to temperature extremes as puparia. This dual advantage may partially explain how quickly *O. biloba* appears to be colonising the UK.

In Scotland, the specimens of the species that were previously thought of as southerly, *O. avicularia* and *O. fringillina*, are from coastal areas, not on the hills like *O. chloropus* that was previously the only species in more northern parts of the UK. Features such as the aspect of a slope or vegetation cover may also produce fine‐scale variation in climatic conditions (de Frenne et al., 2021; Wilson et al., 2015) and areas with suitable microclimates may allow insect species to survive in an otherwise unsuitable area (Minter et al., 2020), as well as allowing some of their bird hosts to achieve a higher reproductive rate (Shutt et al., 2022).

*Ornithomya chloropus* would appear to have a lower minimum thermal tolerance as it is found at higher altitudes than the other species in the same genus and it may be that it also has a lower maximum thermal tolerance as its range has contracted slightly as temperatures have warmed. A similar effect has been observed with some species of the related tsetse flies *Glossina* spp. that are undergoing range contraction in Zimbabwe, where the extinction probability of *G. morsitans* (Westwood, 1851) has been estimated to be a function of temperature (Are & Hargrove, 2020).

Some of the differences in detection of the various species may be due to different sampling methods between the 1960s and the current study. A lot of the results in the older study were from Bird Observatories in the northern half of the UK which may be expected to have more *O. chloropus* than the other species. Apparent changes in the distributions of the generalist *Ornithomya* spp. compared with earlier ranges may in part be due to the inclusion of flies on migrating birds in this study, as Hill removed records he considered accidental, that is those where the host was a passage migrant, from his maps (Hill, 1962a). In the current study, all records were used to produce the maps, but most of the *O. chloropus* on the south coast were on probable migrant species, such as Meadow Pipit *Anthus pratensis* (Linnaeus, 1758), and this may fully explain their predicted occurrences around The Wash and at other localised sites on the south coast in the Maxent SDM maps.

Another major difference is that the current study relied on the capture of flies that left the birds during routine bird ringing: In the 1960s, sampling was usually done using a knock‐down method, with chloroform in a Fair Isle Apparatus (Williamson, 1954), which would have resulted in the removal and capture of a significantly higher proportion, if not all, of the parasites on each bird. Smaller species of fly were generally reported by volunteers to be harder to catch, when relying solely on just the visual acuity and dexterity of the bird ringer, than the larger ones which were easier to see and move more slowly. Larger flies might also be more easily disturbed than smaller ones when a bird is handled and *O. avicularia* is the largest of the three generalist species.

The results of this study, compared with earlier studies of UK louse flies, strongly suggest that the phenology of some of the species has changed. *Ornithomya avicularia* is seen at the same time of year as it was in the 1950s and 1960s (Hill, 1963; Thompson, 1954) as is *O. chloropus* (Corbet, 1956; Hill, 1963) but numbers of both species now peak a month earlier. It may be that the true peak is even earlier, as many volunteers waited until they started seeing louse flies before asking to join the study, resulting in a delay in the start of collecting at the beginning of the season. *Ornithomya fringillina* peaks a month later, and this may be due to its range shift to further north or bias in the previous data sets. For the other three species, there is insufficient data from the study to draw robust conclusions, but it would appear that *S. hirundinis* and *C. pallida* have a similar phenology to that in the mid‐20th century (Hutson, 1981b; Summers, 1975; Thompson, 1953). While resident host species, for example, Great Tit *Parus major* (Linnaeus, 1758), are able to track climate changes and advance their laying date (Charmantier et al., 2008), allowing their parasites to also shift their phenology, the dependence of *S. hirundinis* and *C. pallida* on migrant hosts limits their seasonality. Hirundines are arriving an average of 10 days earlier than in the 1960s but Swifts have not significantly changed their arrival dates (Newson et al., 2016). *Ornithomya biloba* is a recent colonist, with only three UK records before 2007, all from Swallows recently arrived from Africa in May and June, whereas now *O. biloba* breeds in the UK (Wawman, 2024) and is seen from June to October when its host the Swallow is present. However, most of the cited previous studies were small, from single sites or only a few sites, often at Bird Observatories in the northern part of the UK, or used previously published records. It might be possible to produce better comparisons by obtaining all possible original data from publications, museum records and local environmental records centres, but historical data are also biased (Boyd et al., 2022; Guzman et al., 2021; Shirey et al., 2023).

In Fennoscandia, in 2013, the overall prevalence of *O. chloropus* in Pied Flycatcher *Ficedula hypoleuca* (Pallas, 1764) nests was found to be 59% (Eeva et al., 2015), whereas, in this study, only one, louse fly, *O. chloropus*, was collected from a Pied Flycatcher nestling, despite large numbers of Pied Flycatchers being ringed by volunteers, and the author examining over 100 nests for puparia, and monitoring and ringing over 500 broods of Pied Flycatchers in Somerset (Wawman, unpublished data). It may be that by breeding earlier, Pied Flycatchers in the UK miss the peak in louse flies: Pied Flycatchers in Southern Sweden usually start laying eggs in the third week in May (Källander et al., 2017), whereas those in Somerset, UK, may begin laying in late April (Wawman, unpublished data). I hypothesise that *O. chloropus* is a partial nest parasite as an adaptation to the colder climate in more northerly regions.

As species shift their ranges, species that may not have associated are more likely to do so, and for parasites, this may mean switches in host parasite relationships, bringing the risk of diseases to new hosts. The host–parasite associations of the UK Hippoboscidae found in this study will be the subject of further research, but with known vectors of avian disease amongst them, there is the potential for them to have an impact on avian health.

## AUTHOR CONTRIBUTIONS

**Denise C. Wawman:** Conceptualization; investigation; writing – original draft; methodology; validation; visualization; writing – review and editing; formal analysis; project administration; data curation; resources.

## FUNDING INFORMATION

This research received no specific grant from any funding agency, commercial or not‐for‐profit sectors.

## CONFLICT OF INTEREST STATEMENT

The author declares no conflicts of interest.

## Supporting information


**FIGURE S1.** All sites from which louse flies (Hippoboscidae) were collected for the avian louse fly study (all flies excluding keds). UK sites are plotted as white squares at 1 km^2^ resolution and Irish sites in purple.
**FIGURE S2.** Maps showing the distribution of capture sites for all Hippoboscidae in the study (louse flies and keds), as Maxent model outputs predicting the likelihood of a Hippoboscid in the project being collected at a site, relative to (a) the proportion of urban land‐cover in that 1 km^2^, (b) the proportion of protected land‐cover and (c) the proportions of both urban and protected land‐cover. Warmer (redder) colours predict a higher probability that a fly will be found at a site. The areas under the receiver operator curve, and the high proportion of green in these maps indicate that the sites are almost randomly distributed with respect to both urban and protected area land‐cover.
**FIGURE S3.** Kernel densities for all Hippoboscidae in the study. The kernel densities are plotted at 95% predicted probability (dark red), 75% probability and 50% probability of a fly being caught at a given location, during the study, based entirely on latitude, longitude and total count data.
**FIGURE S4.** Boxplots showing the altitude ranges of all of the species of Hippoboscid received during the study period. *Ornithomya chloropus* occurs over a wider altitude range than the other *Ornithomya* sp.

## Data Availability

The dataset for this study is available at https://doi.org/10.5061/dryad.zs7h44jkh. On completion of the study, the records will be entered into the database on the website iRecord with the other Hippoboscidae and Nycteribiidae Recording Scheme data.
