# Supplementary material for: Citizen scientists mapping the United Kingdom's and Republic of Ireland's flat flies (louse flies) (Diptera: Hippoboscidae) reveal a vector's range shift
Source: Med Vet Entomol. 2025 Feb 7;39(3):559–75. doi: 10.1111/mve.12795 (PMC12323749; doi:10.1111/mve.12795)
Supplement: Supplementary file 1 — FIGURE S1. All sites from which louse flies (Hippoboscidae) were collected for the avian louse fly study (all flies excluding keds). UK sites are plotted as white squares at 1 km2 resolution and Irish sites in purple. FIGURE S2. Maps showing the distribution of capture sites for all Hippoboscidae in the study (louse flies and keds), as Maxent model outputs predicting the likelihood of a Hippoboscid in the project being collected at a site, relative to (a) the proportion of urban land‐cover in that 1 km2, (b) the proportion of protected land‐cover and (c) the proportions of both urban and protected land‐cover. Warmer (redder) colours predict a higher probability that a fly will be found at a site. The areas under the receiver operator curve, and the high proportion of green in these maps indicate that the sites are almost randomly distributed with respect to both urban and protected area land‐cover. FIGURE S3. Kernel densities for all Hippoboscidae in the study. The kernel densities are plotted at 95% predicted probability (dark red), 75% probability and 50% probability of a fly being caught at a given location, during the study, based entirely on latitude, longitude and total count data. FIGURE S4. Boxplots showing the altitude ranges of all of the species of Hippoboscid received during the study period. Ornithomya chloropus occurs over a wider altitude range than the other Ornithomya sp. [file MVE-39-559-s001.pdf]

Supporting Information

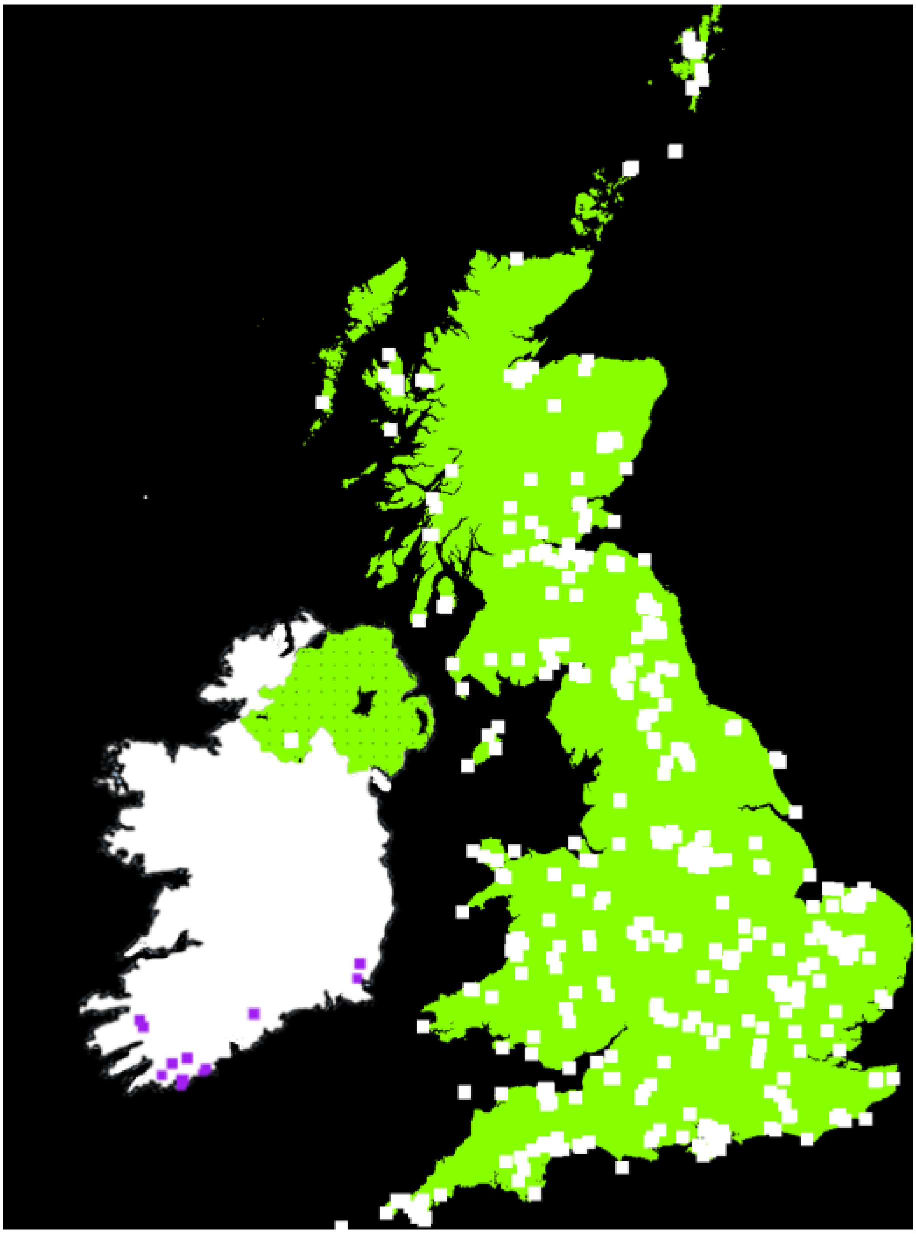

FIGURE S1: All sites from which louse flies (Hippoboscidae) were collected for the avian louse fly study (all flies excluding keds). UK sites are plotted as white squares at 1 km^2 resolution and Irish sites in purple.

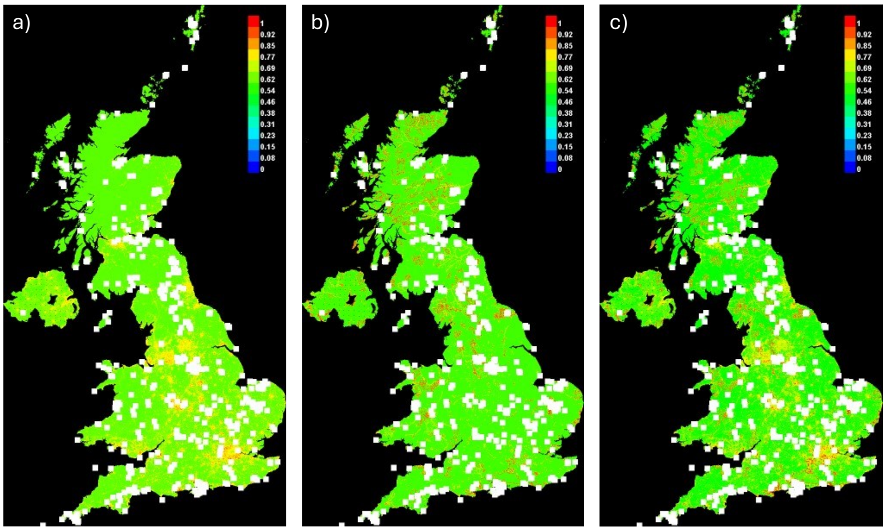

FIGURE S2: Maps showing the distribution of capture sites for all Hippoboscidae in the study (louse flies and keds), as Maxent model outputs predicting the likelihood of a Hippoboscid in the project being collected at a site, relative to a) the proportion of urban land-cover in that 1 km^2, b) the proportion of protected land-cover and c) the proportions of both urban and protected land-cover. Warmer (redder) colours predict a higher probability that a fly will be found at a site. The areas under the receiver operator curve, and the high proportion of green in these maps indicate that the sites are almost randomly distributed with respect to both urban and protected area land-cover.

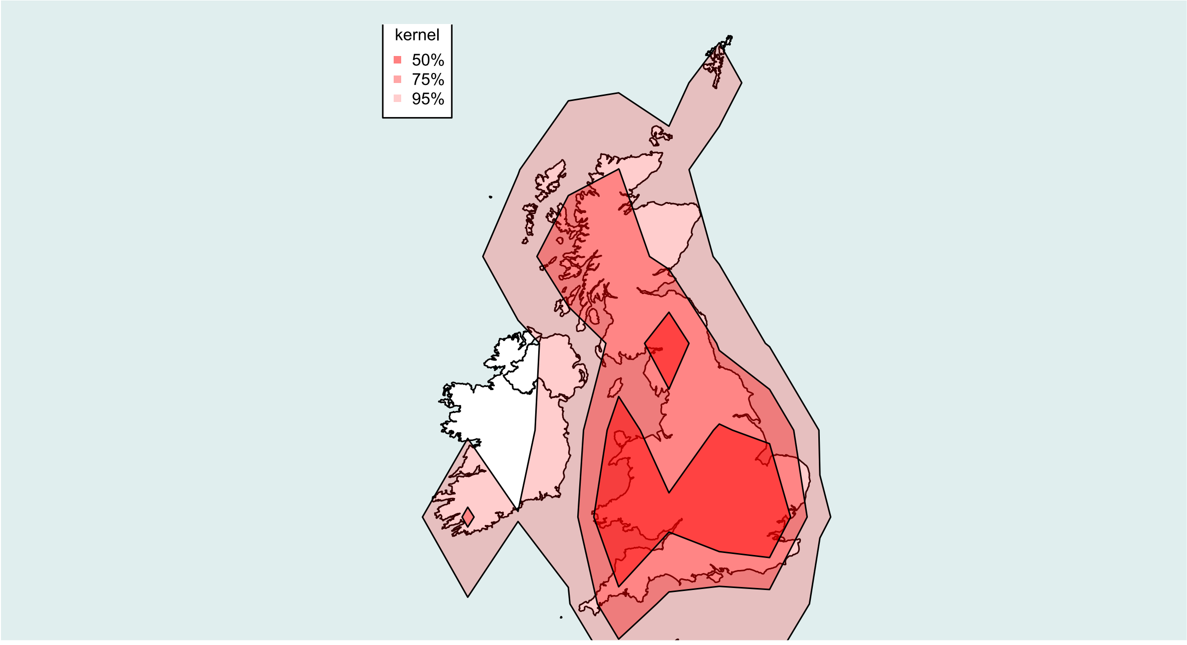

FIGURE S3: Kernel densities for all Hippoboscidae in the study. The kernel densities are plotted at 95% predicted probability (dark red), 75% probability and 50% probability of a fly being caught at a given location, during the study, based entirely on latitude, longitude and total count data.

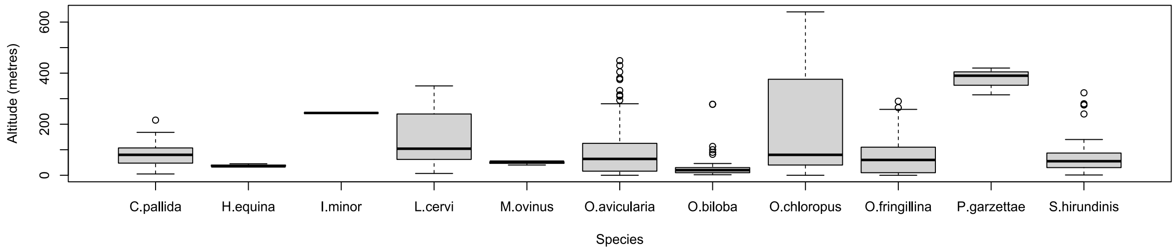

FIGURE S4: Boxplots showing the altitude ranges of all of the species of Hippoboscid received during the study period. *Ornithomya chloropus* occurs over a wider altitude range than the other *Ornithomya sp.*
